# Oral administration of the anti-proliferative substance taurolidine has no impact on dextran sulfate sodium induced colitis-associated carcinogenesis in mice

**DOI:** 10.4103/1477-3163.62536

**Published:** 2010-04-16

**Authors:** Ansgar Michael Chromik, Sebastian Huss, Hayssam Osseili, Adrien Daigeler, Sabine Kersting, Dominique Sülberg, Ulrich Mittelkötter, Thomas Herdegen, Waldemar Uhl, Annette M. Müller

**Affiliations:** Department of General and Visceral Surgery, St. Josef Hospital, Campus Kiel, Germany; # Department of both authors contributed equally to this publication; 1 Department of Pediatric Pathology, University of Bonn, Campus Kiel, Germany; 2 Department of Plastic Surgery, University Hospital Bergmannsheil, Ruhr-University Bochum, Campus Kiel, Germany; 3 Institute of Pharmacology, University Hospital of Schleswig-Holstein, Campus Kiel, Germany

**Keywords:** Carcinogenesis, C57BL6 mice, Dextran Sulfate Sodium, experimental colitis, inflammatory bowel disease, Taurolidin, Taurolin, TRD

## Abstract

**Background:**

New chemopreventive strategies for ulcerative colitis (UC)-associated dysplasia and cancer have to be evaluated. Taurolidine (TRD) has anti-inflammatory, anti-proliferative and anti-neoplastic properties with almost absent toxicity. The aim of the study was to determine whether TRD decreases dysplasia in the well-characterized Dextran Sulfate Sodium – Azoxymethane (DSS-AOM) animal model for UC-associated carcinogenesis.

**Material and Methods:**

The DSS-AOM model of carcinogenesis was induced in female inbred C57BL/6 mice. Half of the mice were treated with TRD, the other served as control. After 100 days macroscopic, histological and immunhistochemical (β-Catenin, E-Cadherin, SOX9, Ki-67, Cyclin-D1) examination of the colon was performed.

**Results:**

Incidence, multiplicity, grading and growth pattern of adenomas did not differ significantly between TRD and control group. In all animals, inflammatory changes were absent. Immunhistochemistry revealed increased expression of Ki-67, β-catenin, SOX9 and Cyclin-D1 in adenomas compared to normal mucosa – without significant difference between TRD and control treatment.

**Conclusion:**

Oral administration of TRD has no impact on DSS-induced colitis-associated carcinogenesis. However, SOX9 and Cyclin-D1 representing key members of the Wnt pathway have not yet been described in the DSS-AOM model of carcinogenesis – underlining the importance of this oncogenic pathway in this setting.

## INTRODUCTION

### Ulcerative colitis-associated colorectal cancer

Patients with ulcerative colitis (UC) have a significantly increased risk of developing colorectal dysplasia and colorectal cancer (CRC) during their lifetime.[1] Although ulcerative colitis-related colorectal cancer accounts for only 1-2% of all CRC cases in the general population, it is responsible for 10-15% of all deaths in UC patients.[2] For the individual UC patient, risk of malignancy is continuously increasing with duration of the disease and therefore several surveillance strategies have been developed.[3] However, in UC patients with a disease history of 30 or more years, the cumulative risk for CRC ranges in recent studies between 10-18%, depending on screening methods, geographic and other epidemiologic factors.[4,5]

### Chemoprevention

Despite surveillance colonoscopy or prophylactic colectomy, the concept of chemoprevention has gained increasing importance during the last years.[6] Many pharmacological agents have been evaluated for their chemopreventive features for UC-associated CRC. The ideal chemopreventive agent would be effective at preventing neoplastic progression, safe (i.e. without side-effects) and inexpensive.[3,6] The most frequently used chemopreventive agents in UC patients are 5-aminosalicylic acid (5-ASA) compounds like mesalazine or sulfasalazine as well as ursodeoxycholic acid (UDCA), which is applied in patients with primary sclerosing cholangitis (PSC).[7] Whereas the chemopreventive role for UDCA in PSC patients is broadly accepted,[8,9] there is still ongoing debate about the chemopreventive capacity of 5-ASA derivates in UC patients without PSC.[3] Although there are numerous studies supporting the chemopreventive efficacy of 5-ASA,[10,11] other authors could not show significant reduction in UC-related CRC or dysplasia.[12,13] As a result, further therapeutic targets and corresponding pharmacological candidates have to be evaluated for chemoprevention in UC-related CRC.

### Taurolidine

Taurolidine (TRD)–a derivate of the aminosulfoacid Taurin – has anti-inflammatory, anti-proliferative and anti-neoplastic properties. So far, TRD was mainly used in the treatment of peritonitis and catheter-related bloodstream infections due to its capacity to inactivate bacterial cell wall components, e.g. lipopolysaccharides and various exotoxins.[14–17] The anti-proliferative and anti-neoplastic effects of TRD have been investigated in several tumor cell lines *in vitro* as well as *in vivo*.[18–21] Inhibition of proliferation and cell death induction by TRD seems to be a multifaceted process and remains to be fully elucidated. However, cell death-inducing mechanisms of TRD include the mitochondrial cytochrome-c-dependent apoptotic pathway,[22,23] inhibition of protein synthesis,[24,25] as well as activation of autophagy.[26] Application of TRD in patients with advanced gastric cancer and glioblastoma showed promising clinical results with almost absent toxicity[27,28] and further oncological trials with iv application of TRD are currently being conducted.

### DSS-AOM model of carcinogenesis

The Dextran Sulfate Sodium – Azoxymethane (DSS-AOM) model is a well-characterized experimental model for UC-associated CRC.[29–33] Mice that are exposed to a single injection of the classic colon carcinogen AOM prior to cyclic administration of DSS in drinking water will develop chronic inflammatory changes[34,35] as well as dysplasia and carcinoma with pathological features that resemble those of human UC-associated neoplasia.[29,32,33] The extent of neoplastic lesions depends on several factors like strain susceptibility as well as duration, dosage and schedule of cyclic DSS application.[36–38]

### Taurolidine as chemopreventive agent?

Besides our experience with TRD as apoptosis-inducing and anti-proliferative agent in colorectal and other cancer cell lines *in vitro*,[19–21] we could recently report that oral application of TRD is well tolerated and significantly ameliorates chronic DSS colitis in C57/BL6 mice.[39] However, the potential role of TRD in chemoprevention of colitis-associated CRC has not been investigated so far. The aim of this study was therefore to evaluate the chemopreventive capacity of oral TRD in the murine DSS-AOM model of carcinogenesis.

## MATERIALS AND METHODS

### DSS-AOM model of carcinogenesis

The DSS-AOM model of carcinogenesis was induced in female inbred C57BL/6 mice (Charles River, Sulzfeld, Germany, body weight 22-25g) which were randomized into two groups with n = 9 animals each. On Day 0, all animals received a single intraperitoneal injection of Azoxymethane (AOM) (Sigma-Aldrich, Munich, Germany) at a dose level of 10 mg/kg body weight and received demineralized water for seven days. Thereafter, a Dextrane sodium sulfate (DSS) colitis (molecular weight 36-44 kDa, MP Biomedicals, Aurora, OH, USA) was induced by a three-cyclic administration of 0.75% DSS (w/v) solution in demineralized water for five days, followed by a five-day DSS-free interval. During the DSS-free interval and after finishing the third DSS cycle (i.e. after 32 days), one group received 0.2% TRD (w/v) in demineralized water (TRD group, n = 9), which has been shown to exert anti-inflammatory effects in previous studies.[39] As control, a second group received demineralized drinking water only (control group, n = 9). After 100 days all animals were killed under deep anesthesia. Mice were housed three per cage with diet and fluids *ad libitum*. During the first 36 days (i.e. induction of DSS colitis), disease activity was quantified every day using the Disease activity index described elsewhere.[34,35,40]

### Histological evaluation of H and E sections

After sacrificing the mice after 100 days, the colon was removed and rinsed with normal saline. The length of the colon was measured and macroscopic photographs were taken. Thereafter, the colon was divided into four parts, i.e. cecum, proximal, mid and distal colon and fixed in 10% buffered formaline for 24 h. Histological examination of the entire colon was performed on paraffin-embedded sections after H and E staining. Histological slides were evaluated independently by two investigators (S.H. and A.M.M.) blinded to the respective groups. Proliferative colonic mucosal changes e.g. hyperplasia, aberrant crypt foci (ACF), gastrointestinal intraepithelial neoplasia (GIN), adenoma and adenocarcinoma were diagnosed according to the *Pathology of Mouse Models of Intestinal Cancer: Consensus Report and Recommendations*.[30] With respect to inter-observer agreement, differences were found in less than 5% of observations. Any differences in grading were resolved by joint examination.

### Immunohistochemistry

Immunohistochemistry (IHC) for β-catenin, E-Cadherin, SOX9, Ki- 67, and Cyclin-D1 was performed on 4-*μ*m-thick paraffin-embedded sections by use of the peroxidase-conjugated avidin-biotin method. Deparaffinized and rehydrated sections were incubated with the following primary antibodies: anti-β-catenin (1:1000, Transduction Lab., Lexington, UK), anti-E-Cadherin (1:50, Santa Cruz, Europe), anti-Ki- 67 (1:25, DAKO, Germany), anti-Cyclin-D1 (1:25, DCS, Hamburg, Germany) and anti-SOX9 (1: 500, RnD Systems, Germany). Immunoreactions were visualized by using 3-amino-9-ethylcarbazole (AEC) as a substrate (DAKO Real Detection System, Ref: K5003, Germany) following incubation with biotinylated secondary antibodies. Based on the immunoreactivity score (IRS) by Remmele a scoring system was used to describe the staining intensity (negative, weak, moderate, and strong) and proportion (0-100%) of cells stained.[41] Integer values were assigned to intensity scores (0-3) and proportion of stained cells and multiplied to provide a single integrated score for each staining. The data were reduced to an ordinal scale of 0 to 3. For β-catenin three different cell localizations, nuclear (N), cytoplasmic (C) and membrane (M) were analyzed. For the estimation of nuclear staining by Cyclin-D1 and Ki-67, the percentage of stained nuclei were scored and integer values were assigned to the proportion of cells stained (0-4).[42] All histological quantifications were first performed as independent analysis by two observers (S.H and A.M.M.) as described above. Any discrepancies were resolved by simultaneous reevaluation of the sections by both observers using a multiheaded microscope.

### Statement of animal care

All animal experiments have been performed according to the German law for protection of animals and the NIH guidelines for use and care of laboratory animals. All experiments were approved by the Ministerium fuer Landwirtschaft und Naturschutz, Kiel, Germany (V742-72241.121-22 (19-2/05)).

### Statistical evaluation

Results for disease activity index (DAI) and immunoreactivity score (IRS) were expressed as means ± SEM. Colon length was displayed in Box-Whisker plots indicating median and 5^th^/95^th^ percentiles. DAI values were analyzed using repeated measures analysis of variance (ANOVA) over all time points. Incidence, grading and growth pattern of adenomas were compared by Fisher's exact test between both groups, whereas multiplicity of adenomas was illustrated by scatter plot and tested by Mann Whitney test. IRS was analyzed by Kruskal-Wallis test and Dunn's multiple Comparison test. Comparison of colon lengths between both groups was performed by Mann Whitney test. *P* values ≤ 0.05 were considered statistically significant and indicated with asterixes (* *P* ≤ 0.05; ** *P* ≤ 0.01; *P* *** ≤ 0.001).

## RESULTS

### Impact of Taurolidine on disease activity

**Figure 1:**
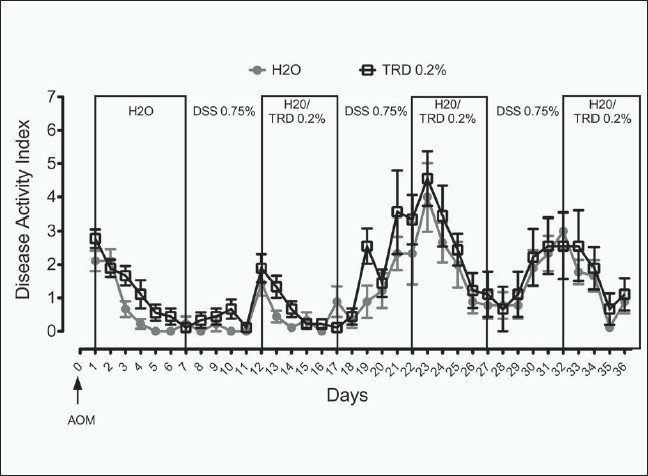
Disease Activity Index (DAI) of mice subjected to the DSSAOM (Dextran Sulfate Sodium - Azoxymethane) model of carcinogenesis after treatment with 0.2% Taurolidine (TRD) during DSS-free intervals compared to control treatment with water (H_2_O) (each n = 9). There was a tri-phasic colitis with peak DAI values at the end of each DSS treatment interval (i.e. Day 12, 22 and 32, respectively) without any significant differences between the TRD group (TRD 0.2%) and the control group (H_2_O) (repeated measures ANOVA over all time points). After 100 days, survival was 100% in both groups.

As indicated in Figure 1, application of AOM (10 mg/kg body weight) followed by cyclic administration of 0.75% DSS resulted in a tri-phasic colitis with peak DAI values at the end of each DSS treatment interval (i.e. Day 12, 22 and 32, respectively) and recovery during the DSS-free interval [Figure 1]. There were no significant differences in DAI values between the TRD group (TRD 0.2%) and the control group (H_2_O) during this induction period of 37 days (repeated measures ANOVA over all time points) [Figure 1]. After 100 days, survival was 100% in both groups.

### Macroscopic and microscopic evaluation

**Figure 2:**
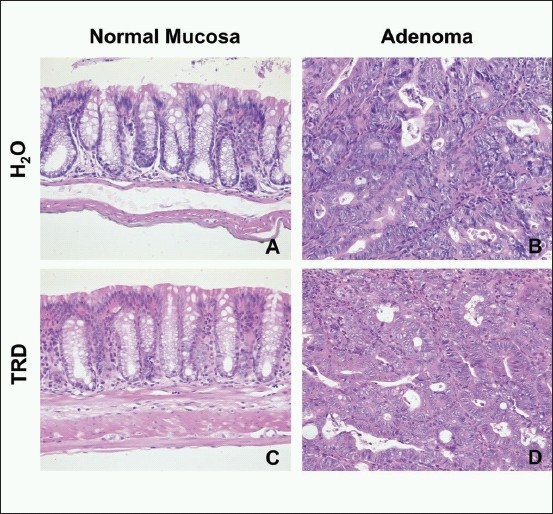
Representative histological Hematoxylin and Eosin (H and E) staining of normal colonic mucosa (A, C) and adenoma (B, D) in mice subjected to the DSS-AOM (Dextran Sulfate Sodium - Azoxymethane) model of carcinogenesis and treated with Taurolidine (TRD) 0.2% or drinking water as control (H_2_O). A+C: normal colonic mucosa with regular crypt architecture; B+D: tubular adenoma with mild dysplasia (low grade) (×200 magnification). No difference between TRD and control treatment

The colon length did not differ significantly between the TRD group (mean 9.1 cm ± 0.2 cm SEM) and the control group (mean 9.1cm ± 0.3cm SEM) (Mann Whitney test). In the control group, 6/9 animals developed adenoma leading to an incidence of 66.7% compared to 5/9 animals (55.6%) in the TRD group, which was not significantly different (Fisher's exact test). The multiplicity of adenomas was also not affected by TRD application, since the median number of adenomas per animal between control group (1; range 0-7) and TRD group (2; range 0-4) was not significantly different (Mann Whitney test). In the TRD group as well as in the control group (H_2_O), the majority of adenomas was low-grade adenoma (73% vs. 78%) and had a tubular growth pattern (80% vs. 89%) without any significant differences between TRD and control group (Fisher's exact test). In all animals, inflammatory changes (e.g. mucosal ulceration, crypt loss), aberrant crypt foci (ACF) or adenocarcinoma were absent. Representative histological H and E staining for normal mucosa and tubular adenoma (low grade) of the TRD group and control group (H_2_O) are provided in Figure 2.

### Immunoreactivity scores

**Figure 3:**
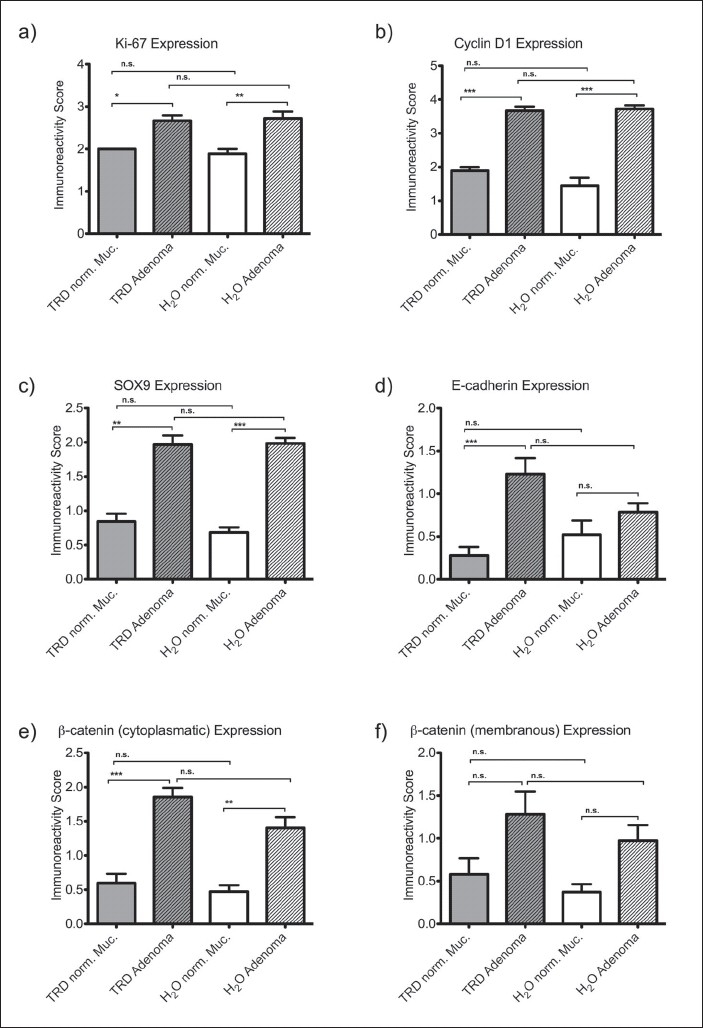
Immunoreactivity scores (IRS) for Ki-67 (a), Cyclin-D1 (b), SOX9 (c), E-cadherin (d) as well as cytoplasmatic (e) and membranous β-catenin (f) in colonic adenoma and normal colonic mucosa of mice subjected to the DSS-AOM model of carcinogenesis and treated with Taurolidine (TRD) and water in the control group (H_2_O). Bars indicate mean ± SEM, statistical analysis was carried out by Kruskal-Wallis test and Dunn's Multiple Comparison test (*P≤0.05; **P≤0.01; P***≤0.001)

After induction of the DSS-AOM model of carcinogenesis (i.e. after 100 days), colonic adenomas as well as normal colonic mucosa were analyzed by immunostaining for different antigens known to be associated with the colitis dysplasia-carcinoma sequence. Immunoreactivity results were compared between adenoma and normal mucosa as well as between TRD-treated animals (TRD) and the control group (H_2_O) [Figure 3].

The expression of the proliferation marker Ki-67 was characterized by a significantly higher expression in adenomatous tissue compared to normal mucosa. Immuoreactivity score (IRS) for Ki-67 in the control group (H_2_O) was 2.7 (± 0.2) in adenoma compared to 1.9 (± 0.1) in normal mucosa (*P* ≤ 0.01). In the TRD group (TRD), Ki-67 score in adenomatous tissue was 2.7 (± 0.1) and in normal mucosa 2.0 (± 0.0) (*P* ≤ 0.05). There was no significant difference between control group (H_2_O) and TRD treatment (TRD) in Ki-67 expression [Figure 3a].

The cell cycle regulator Cyclin-D1 and the transcription factor SOX9 displayed a significantly increased expression in adenomas compared to normal mucosa [Figure 3b, c]. IRS for Cyclin-D1 in adenomas compared to normal mucosa was 3.7 (± 0.1) vs. 1.4 (± 0.2) in the control group (H_2_O) compared to 3.7 (± 0.1) vs. 1.9 (± 0.1) in the TRD group (*P* ≤ 0.001) [Figure 3b]. The overall expression of SOX9 was less pronounced, leading to IRS between 0.7 to 2.0 as indicated in Figure 3c. IRS for SOX9 in adenomas compared to normal mucosa was 1.9 (± 0.1) vs. 0.7 (± 0.1) in the control group (H_2_O) (*P* ≤ 0.01) compared to 2.0 (± 0.1) vs. 0.8 (± 0.1) in the TRD group (*P* ≤ 0.001) [Figure 3c]. Again, there were no significant differences for both parameters between TRD and control treatment [Figure 3b, c].

E-cadherin showed a heterogeneous expression among both treatment groups [Figure 3d]. Only in the TRD group, IRS for E-cadherin was significantly increased in adenoma (1.2 ± 0.2) compared to normal mucosa (0.3 ± 0.1) (*P* ≤ 0.001) whereas IRS in the control group (H_2_O) did not differ significantly between adenoma (0.8 ± 0.1) and normal mucosa (0.5 ± 0.2) [Figure 3d].

The degree of β-catenin immunoreactivity was highly dependent on its intracellular localization, since β-catenin was only detectable in the cytoplasm [Figure 3e] and in the cell membrane [Figure 3f]. There was no staining of the nucleus. Cytoplasmatic β-catenin was significantly increased in adenomas compared to normal mucosa leading to an IRS of 1.4 (± 0.2) vs. 0.5 (± 0.1) in the control group (H_2_O) (*P* ≤ 0.001) and 1.9 (± 0.1) vs. 0.6 (± 0.1) in the TRD group (*P* ≤ 0.01) without any significant differences between TRD and control treatment [Figure 3e]. Membranous β-catenin showed a similar expression pattern with elevated IRS in adenomas compared to normal mucosa. However, the differences did not reach statistical significance [Figure 3f].

### Tissue compartment specific results of immunostaining

**Figure 4:**
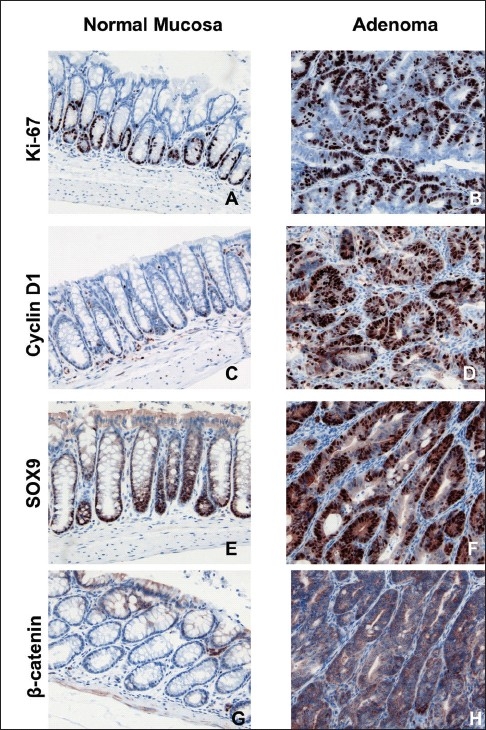
Representative immunhistochemistry staining of Ki-67, Cyclin-D1, SOX9 and β-catenin of normal colonic mucosa (left side) and adenoma (right side) in mice subjected to the DSS-AOM (Dextran Sulfate Sodium - Azoxymethane) model of carcinogenesis without treatment (control group) (x200 magnification)

Figure 4 displays representative immunostaining of antigens analyzed in this study. Since there were no significant differences in antigen expression between the TRD group and the control treatment, only photographs of the control group (H_2_O) are presented.

In normal mucosa, Ki-67 staining was restricted to the lower part of the crpyts, whereas adenomas showed a strong staining of the majority of the cells [Figure 4a, b]. Similarly, Cyclin-D1 and SOX9 immunostaining in normal mucosa was characterized by localized expression in the basal part of the crypts, representing the proliferating compartment [Figure 4c, e]. In contrast, adenomatous tissue displayed a strong immunostaining in the majority of cells [Figure 4d, f]. The cytoplasmatic expression of β-catenin in normal mucosa was limited to scattered superficial cells, whereas adenoma cells showed a pronounced cytoplasmatic staining [Figure 4g, h].

## DISCUSSION

In this study, we sought to determine the chemopreventive capacity of oral TRD – a derivate of the aminosulfoacid Taurine – in the DSS-AOM model of carcinogenesis. The rationale for this study was based on two unique characteristics of TRD: its anti-neoplastic and anti-inflammatory properties. TRD has been shown to mediate anti-proliferative and anti-neoplastic effects towards different colon carcinoma cell lines *in vitro*[19,24,43–47] as well as *in vivo* in different animal models of colon carcinoma with i.p.[44–46,48–53] or i.v. application[54–58] of TRD. Recently, i.p.-applicated TRD has been evaluated in patients undergoing major surgery for CRC. TRD was responsible for reducing cytokines like IL- 1β which are regarded as “tumor-stimulating cytokines” – representing a surrogate parameter for metastasis formation.[59] Furthermore, i.v. application of TRD in patients with advanced gastric cancer and glioblastoma showed promising clinical results with almost absent toxicity[27,28] and further oncological trials with i.v. application of TRD are currently being conducted. The anti-inflammatory action of TRD is caused by its ability to inactivate lipopolysaccharides or exotoxins and to inhibit secretion of different cytokines like TNFα, IL-1 and IL-8, which has been demonstrated *in vitro*[60,61] and *in vivo*.[62–64] This led to a broad spectrum of clinical applications, e.g. prevention and treatment of central venous catheter-related bloodstream infections[16,65] or treatment of peritonitis.[66–68] In a previous study we could recently report that oral application of TRD significantly attenuates chronic DSS colitis in mice leading to significantly reduced disease activity and mortality.[39] Those results were consistent with the finding provided by another group, that i.v. application of TRD reduces disease activity and endotoxemia in the hapten induced TNBS colitis model in mice.[69]

However, in the DSS-AOM model of carcinogenesis we could not find any chemopreventive effect of TRD. Oral application of 0.2% TRD did not demonstrate any significant chemopreventive effect in terms of incidence, multiplicity, grading or growth pattern of adenoma in the DSS-AOM model of carcinogenesis. Furthermore, there was no difference in the expression of the proliferation marker Ki-67 or the expression of different members of the Wnt signaling pathway (Cyclin-D1, β-catenin, E-cadherin or SOX9) between TRD-treated animals and the control group. Considering the strong clinical amelioration we encountered previously with oral application of TRD 0.2% in the chronic DSS colitis model,[39] one reason for the missing chemopreventive effect could be the oral application of TRD, that might not be sufficient to exert anti-proliferative activity. Another reason could be that TRD interferes with inflammatory cytokines that are sometimes active in malignant tumors. In contrast to such tumors as well as in contrast to severe colitis, after 100 days, here, the disease activity as well as aberrant crypt foci were absent. We regard the chemopreventive effect of TRD as less relevant as it could be expected from previous studies.[39]

Besides studying the chemopreventive effect of TRD in the DSS-AOM model of carcinogenesis, we tried to gain further insight into the experimental model itself.[32] Hence, we analyzed by immunohistochemistry some components of the Wnt signal transduction pathway, which has been identified as one of the key pathways in the initiation and development of CRC,[70,71] as well as in the DSS-AOM model of carcinogenesis.[72–75] β-catenin – a crucial downstream effector of the Wnt signaling pathway – showed a significantly higher cytoplasmatic expression in adenomas compared to normal mucosa whereas the membranous expression was almost unaffected by the application of DSS-AOM. This observation is supported by other authors, who similarly encountered a cytosolic accumulation of β-catenin in adenomas as well as in adenocarcinomas using the same experimental model.[73,75] In human colon cancer, the cytosolic accumulation of β-catenin is either caused by mutational inactivation of the AP C tumor suppressor gene or by mutation of β-catenin itself leading to nuclear translocation and binding to TCF4 (T-cell Factor 4). The β-catenin/TCF4 complex activates transcription of several oncogenic genes.[70,76] We analyzed two important target genes of the β-catenin/TCF4 complex that have not been described so far in the DSS-AOM model: Cyclin-D1 and SOX9. Cyclin-D1 represents a pivotal regulator of cell cycle progression since Cyclin-D1 facilitates transcription of various genes that are required for the transition from the G1- to S-phase and for DNA replication. Therefore, overexpression of Cyclin-D1 has been linked to the development and progression of cancer.[77,78] So far, only one study has focused on the expression of Cyclin-D1 in the DSS-AOM model and the expression profile was assessed in integrin-linked kinase knockout mice and not compared to normal mucosa.[79] Our study describes for the first time a strong increase in the expression of Cyclin-D1 in adenomas compared to normal tissue in the DSS-AOM model of carcinogenesis – supporting the importance of the Cyclin-D1 and the Wnt pathway in this experimental setting. SOX9 represents another effector of Wnt signaling and important target gene of β-catenin in the intestine.[80–82] SOX9 is a member of the SOX (SRY box = sex determining region Y box) gene superfamily of transcription factors and is characterized by a highly conserved high-mobility group (HMG) DNA-binding domain. SOX9 has been shown to be involved in the development and differentiation of many cell types and tissues, e.g. chondrocytes,[83] pancreatic tissue,[84] prostate,[85] testis[86] as well as melanocytes.[87] Furthermore, SOX9 is crucial for the development of intestinal epithelium since inactivation of SOX9 results in severe defects in differentiation of Paneth and Globlet cells.[88] There is growing evidence that SOX9 also plays an important role in different malignancies e.g. prostate, brain or breast cancer.[89–91] Recently, it has been shown that SOX9 is highly overexpressed in CRC[92] and overexpression is significantly associated with a lower five-year survival.[93] So far SOX9 expression has neither been reported in human UC-associated CRC or animal models like the DSS-AOM model of carcinogenesis. In our study, adenomas were characterized by significantly higher SOX9 expression compared to normal mucosa suggesting an important role in this paradigm of UC-associated CRC.

## CONCLUSIONS

The anti-inflammatory and anti-neoplastic substance Taurolidine did not show any chemopreventive capacity in the DSS-AOM model of carcinogenesis. There was no difference in adenoma formation and biology between TRD-treated animals and untreated animals. However, the expression of SOX9 and Cyclin-D1 – two key players of the Wnt signaling pathway – has been described for the first time in this model for UC-associated CRC.

## AUTHOR'S PROFILE

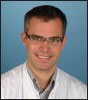



**Dr. Ansgar Michael Chromik** PERSONAL DETAILS Name Ansgar Michael Chromik, MD Date of birth September 5th, 1974 Place of birth Kiel, Germany FIELDS OF INTEREST Research: Apoptosis, surgical oncology, Inflammatory bowel disease, sepsis, clinical ethics consultation CURRENT POSITIONS Since 2007 Attending Surgeon in the Dep. of Genereal and Visceral Surgery, St. Josef-Hospital, Ruhr University of Bochum Germany PREVIOUS POSITIONS 2004-2007 Residency in Genereal Surgery at the Surgical Department, St. Josef-Hospital, Ruhr University of Bochum Germany 2002-2004 Residency in General Surgery at the Surgical Deparment, University Hospital of Münster, Germany EDUCATION 2007 Board Certification in “General Surgery” 2003 Doctoral degree “magna cum laude” in medicine, University of Kiel, Germany 2002 Final examination in medicine at the Humboldt University of Berlin, Germany 2002 USMLE, Step II.
